# Mississippi Valley Medical Association

**Published:** 1901-01

**Authors:** 


### Mississippi Valley Medical Association.

PLACE OF MEETING, PUT IN BAY, OHIO.

OFFICERS.

*President*—A. H. Cordier....................Kansas City, Mo.
*Vice-Presidents*—C. F. McGahan..................Aiken, S. C.
Charles L. Minor..................Asheville.
*Secretary*—Henry E. Tuley....................Louisville, Ky.
*Treasurer*—Dudley S. Reynolds................Louisville, Ky.
*Chairman of Committee of arrangements*..................
J. C. Culberson.............................Cincinnati.

